# The Multifaceted Roles of Primary Cilia in the Development of the Cerebral Cortex

**DOI:** 10.3389/fcell.2021.630161

**Published:** 2021-02-02

**Authors:** Kerstin Hasenpusch-Theil, Thomas Theil

**Affiliations:** ^1^Centre for Discovery Brain Sciences, University of Edinburgh, Edinburgh, United Kingdom; ^2^Simons Initiative for the Developing Brain, University of Edinburgh, Edinburgh, United Kingdom

**Keywords:** primary cilium, cerebral cortex, ciliopathy, signaling, radial glial cells, corpus callosum

## Abstract

The primary cilium, a microtubule based organelle protruding from the cell surface and acting as an antenna in multiple signaling pathways, takes center stage in the formation of the cerebral cortex, the part of the brain that performs highly complex neural tasks and confers humans with their unique cognitive capabilities. These activities require dozens of different types of neurons that are interconnected in complex ways. Due to this complexity, corticogenesis has been regarded as one of the most complex developmental processes and cortical malformations underlie a number of neurodevelopmental disorders such as intellectual disability, autism spectrum disorders, and epilepsy. Cortical development involves several steps controlled by cell–cell signaling. In fact, recent findings have implicated cilia in diverse processes such as neurogenesis, neuronal migration, axon pathfinding, and circuit formation in the developing cortex. Here, we will review recent advances on the multiple roles of cilia during cortex formation and will discuss the implications for a better understanding of the disease mechanisms underlying neurodevelopmental disorders.

## Introduction

The cerebral cortex contains dozens of different types of neurons (van den Ameele et al., 2014). This diversity is thought to underlie its ability to mediate our intellectual capabilities. Therefore, the mechanisms underlying cortical development are at the core of what makes us humans (van den Ameele et al., 2014) and identifying such mechanisms is of central interest in Developmental Neurobiology. Corticogenesis itself represents a multi-step process that initially involves the subdivision of the telencephalon into distinct dorsal and ventral domains giving rise to the neocortex and the basal ganglia, respectively. This patterning process is accompanied by an expansion of radial glial cells, neural stem cells, that eventually undergo neurogenesis to form cortical projection neurons. Newly born neurons leave the stem cell niche and migrate radially along the radial glial fibers to their final position in the cortical plate where they mature forming dendrites and axonal connections. These diverse steps are controlled by multiple signaling events. Signals from within and from outside the cortex control patterning, the formation of neurons, their migration and their integration into neuronal networks to eventually enable cortical functioning. Given this importance of cell signaling, elucidating the roles of primary cilia in cortical development has gained much attention in recent years.

Primary cilia are small protrusions from the cell surface and act as signaling hubs in neural development. Harboring the cell surface receptors for multiple signaling pathways, this tiny organelle receives the signals controlling corticogenesis and converts them into cellular responses, for example into changes in gene expression and/or alterations in the migratory behavior of cells and axons. Astonishingly, the cilium can even influence axon pathfinding decisions by the axonal growth cone which is separated from the cilium by large distances, in case of the corticospinal motor neurons by several centimeters (Guo et al., 2019). Moreover, defects in the function and/or structure of primary cilia underlie a group of syndromes commonly referred to as ciliopathies. There are at least 35 human syndromes in which primary cilia are affected with an increasing number of established and candidate ciliopathy-associated genes (Reiter and Leroux, 2017). Ciliopathies are characterized by pleiotropic clinical features and many ciliopathy patients display severe neurological symptoms, most commonly intellectual disability (ID) and ataxia that often coincide with brain malformations including agenesis of the corpus callosum, abnormal brain size (microcephaly/macrocephaly), hydrocephalus and hypothalamic hamartomas (Valente et al., 2014) but little is known about the underlying disease mechanisms. In turn, many candidate genes for autism spectrum disorder (ASD), schizophrenia, and ID affect primary cilia function (Lee and Gleeson, 2011; Louvi and Grove, 2011; Marley and von Zastrow, 2012; Guo et al., 2017) thereby linking cilia to other neurodevelopmental disorders not commonly regarded as classical ciliopathies. In this review, we briefly describe the structural features of primary cilia and illustrate how they control cell signaling in the cortex. We then discuss the involvement of primary cilia in neurogenesis, neuron migration and neuronal circuit formation during corticogenesis. With respect to cortical patterning, we would like to refer the interested reader to an excellent recent review (Park et al., 2019).

## Primary Cilia, Cell–Cell Signaling and Cortical Development

**FIGURE 1:**
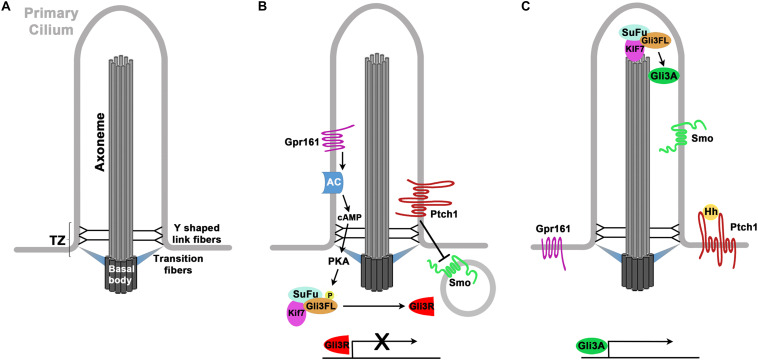
Structure of the cilium and Hedgehog signaling. **(A)** Structure of the primary cilium. Microtubuli are emanating from the basal body in a 9 + 0 arrangement thereby forming the axoneme. The cilium is separated from the cytoplasm and the plasma membrane by the transition zone which consists of the transition and Y shaped link fibers. **(B,C)** Primary cilia control Hedgehog (Hh) signaling. **(B)** In the absence of Hh proteins, Patched (Ptch1) inhibits the ciliary entry of Smoothened (Smo). Gpr161 activity leads to increased formation of cAMP, activation of Protein Kinase A (PKA) and phosphorylation of Gli3 full length (Gli3FL). Gli3FL becomes proteolytically cleaved at the ciliary base to form the Gli3 repressor (GLI3R) that represses the expression of Hh target genes. **(C)** Upon binding of Hh protein, Ptch1 can no longer inhibit ciliary entry of Smo. A complex of Kif7, Sufu and Gli3FL accumulates at the ciliary tip where an activation of GLI3FL occurs. The resulting Gli3 activator protein (Gli3A) activates the expression of Hedgehog target genes.

Primary cilia are microtubule based cell organelles that function as sensors for chemical and mechanical cues from the cellular environment. Nine outer microtubule doublets emanate from the basal body at the ciliary base to form the ciliary axoneme which protrudes from the cell surface (Figure 1) (Fisch and Dupuis-Williams, 2011). Primary cilia lack a central microtubule pair in a 9 + 0 arrangement. A transition zone acts as a filter to control the entry and exit of proteins in and out of the cilium, respectively (Garcia-Gonzalo et al., 2011; Reiter et al., 2012). Located at the base of the axoneme, the transition zone (TZ) seals the cilium from the cytoplasm. Thereby the cilium forms a separate cell compartment with a distinct protein and membrane composition. Within the cilium, proteins are transported using the intraflagellar transport (IFT) machinery (Mourao et al., 2016). The IFT-B complex moves cargo in the anterograde direction from the base of the cilium to the ciliary tip using Kinesin motor proteins while retrograde transport from the tip toward the ciliary base involves IFT-A complexes and dynein motors (Ishikawa and Marshall, 2017).

### Primary Cilia, Hedgehog Signaling and Cortical Development

Primary cilia play pivotal roles in several signaling pathways including Notch/Delta, canonical and non-canonical Wnt, receptor tyrosine kinase and mTOR signaling that are critical for proper cortical development (Park et al., 2019). At the molecular level, ciliary roles are best understood for the Hedgehog (Hh) signal transduction pathway which shall be briefly outlined here. In the absence of signal, the Hh receptor Patched1 (Ptch1) is localized to the cilium and prevents the Hh signal transducer Smoothened (Smo) from accumulating in the cilium (Figure 1B) (Rohatgi et al., 2007). Moreover, the negative regulator Gpr161 activates Protein Kinase A (PKA) (Mukhopadhyay et al., 2013) that in turn phosphorylates the Gli3 transcription factor. As a consequence, Gli3 becomes proteolytically cleaved by the proteasome at the base of the cilium in a ciliary dependent manner to form the Gli3 repressor (Gli3R) (Haycraft et al., 2005) which represses the activity of Hh target genes. Upon binding of Hh proteins, Ptch1 is endocytosed and this removal promotes the enrichment and activation of Smo in the cilium (Figure 1C) (Corbit et al., 2005; Rohatgi et al., 2007; Kopinke et al., 2020). Moreover, Sufu, Kif7, and Gli2 and Gli3 accumulate at the tip of the cilium (Corbit et al., 2005; Haycraft et al., 2005; Liem et al., 2009; Wen et al., 2010) where another phosphorylation event converts the full length Gli proteins into transcriptional activators (GliA) (Han et al., 2019) which in turn activate the expression of Hh target genes. Hence, primary cilia have a dual role in generating the Gli repressor and activator forms and thereby control the GliR/GliA ratio which is crucial for controlling the development of many organ systems including most parts of the neural tube (Persson et al., 2002). Based on several observations, however, development of the cerebral cortex largely depends on the Gli3 repressor but not on the GliR/GliA balance. To date, no cortical malformations have been reported in *Gli2* mutant mice while *Gli3* null mutants present with severe morphological and molecular defects in the dorsal telencephalon (Johnson, 1967; Franz, 1994; Grove et al., 1998; Theil et al., 1999). In contrast to other parts of the developing nervous system, these abnormalities in the developing *Gli3* mutant cortex are not rescued in *Shh*;*Gli3* double mutants (Rallu et al., 2002; Rash and Grove, 2007) suggesting that in addition to its role in suppressing Shh signaling *Gli3* has Shh independent roles in cortical development. Such roles likely include controlling the expression of Wnt/Bmp and Fgf signaling molecules from the cortical hem and the commissural plate, respectively (Grove et al., 1998; Theil et al., 1999). Consistent with this idea, the Gli3^Δ699/Δ699^ mouse mutant, which can only form the repressor but not the Gli3 activator, shows no obvious cortical defects (Böse et al., 2002). Taken together with the lack of a *Gli2* mutant phenotype this finding suggests that Gli activator function is dispensable for normal cortical development. Moreover and as will be discussed later, re-introducing the Gli3 repressor form efficiently rescues patterning and stem cell defects of ciliary mouse mutants (Besse et al., 2011; Hasenpusch-Theil et al., 2020). Hence, it appears that under physiological conditions primary cilia mainly function in cortical development by controlling the levels of the Gli3 repressor rather than by regulating the balance between Gli3 repressor and activator. Gli activator functions may only become obvious when mutations in Hh signaling components result in Hh derepression and pathway hyperactivation (Kopinke et al., 2020).

## Primary Cilia in Cortical Stem and Progenitor Cells

**FIGURE 2:**
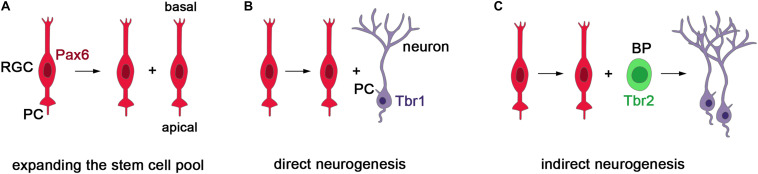
Stem and progenitor cells in the developing mouse cerebral cortex. **(A)** Radial glial cells (RGCs) represent the major neural stem cell type, express the Pax6 transcription factor, project a cilium into the ventricular lumen and divide at the ventricular surface (Götz et al., 1998; Warren et al., 1999). Initially, RGCs divide symmetrically to produce two daughter radial progenitors that help to expand the progenitor pool. **(B,C)** As neurogenesis begins, asymmetric divisions of RGCs produce an RGC daughter cell and either a neuron or a basal progenitor (BP). BPs delaminate from the ventricular surface and are located more basally. They express the Tbr2 transcription factor and mainly undergo symmetric neurogenic divisions predominately producing neurons of the upper cortical layers (Englund et al., 2005). In this way, RGCs can produce neurons either directly or indirectly via the generation of BPs. Importantly, the balance between the symmetric and asymmetric division modes need to be tightly controlled to allow for the formation of appropriate numbers of neurons.

In several ciliopathies, patients present with abnormal cortical growth such as ventriculomegaly, macrocephaly, or microcephaly (Budny et al., 2006; Davis et al., 2007; Putoux et al., 2011; Bachmann-Gagescu et al., 2012; Jamsheer et al., 2012). These malformations and the role of cell-cell signaling in governing the cell lineage of cortical stem and progenitor cells (Seuntjens et al., 2009; Parthasarathy et al., 2014; Cardenas et al., 2018) strongly suggest that primary cilia are crucial for controlling cortical growth. The study of ciliary mouse mutants are supportive of this idea. During murine corticogenesis, there are two main types of progenitor cells: radial glial cells (RGCs) which are neural stem cells and basal progenitors (BPs) that work as transit amplifying cells to increase the neuronal output. RGCs can divide symmetrically to expand the stem cell pool or asymmetrically to produce neurons either directly or indirectly via BPs (Figure 2). The balance between these division modes needs to be tightly controlled to form a cortex of the appropriate size.

A number of electron microscopy studies and immunofluorescence analyses have identified the primary cilium in radial glial cells protruding from the apical membrane into the ventricular lumen. In this way, the cilium is strategically placed to detect cell fate determinants in the ventricular fluid important for controlling the balance between proliferative and differentiative divisions (Wilsch-Brauninger et al., 2012; Paridaen et al., 2013). In addition, the cilium may play an active role in determining the stem cell fate of RGC daughter cells (Paridaen et al., 2013). As in other proliferating cells, primary cilia are assembled during the G1 phase of the cell cycle and are disassembled before mitosis. However, this disassembly is not complete and a ciliary membrane remnant remains attached to the mother centriole. The cell that inherits the ciliary membrane is significantly more likely to retain stem cell character, re-assembles the cilium faster and is capable of responding to external signals quicker than its sister cell. It has therefore been proposed that asymmetric inheritance of the ciliary remnant influences cell fate determination of daughter cells (Paridaen et al., 2013) but it remains to be seen whether mutations in ciliary genes affect the inheritance of the ciliary remnant and thereby cell fate decisions.

Interestingly, nascent basal progenitor cells assemble their cilium after mitosis in the basolateral rather than in the apical plasma membrane even before they delaminate from the ventricular surface (Wilsch-Brauninger et al., 2012). A detailed electron microscopy study revealed that most of these basolateral cilia were embedded in a ciliary pocket with a small opening to the intercellular space whereas others fully protruded into the intercellular space. Given their location, the basolateral cilia cannot detect signals in the ventricular fluid unlike apical cilia but are exposed to signals present in the intercellular space and/or originating from the lateral plasma membrane of adjacent cells. This different exposure to signals may therefore contribute to the cell fate changes associated with the differentiation into basal progenitors (Wilsch-Brauninger et al., 2012). However, the consequences of inactivating basolateral cilia in basal progenitors and the identity of signals to which these cilia respond remain to be determined.

In contrast, ciliary functions in RGCs and the associated signaling pathways have extensively been investigated. Analyses of several ciliary mouse mutants revealed a variety of RGC phenotypes ranging from severe effects on proliferation to more subtle effects in the balance between direct and indirect neurogenesis.

### Effects of Depleting Cilia on Cortex Development

Several groups addressed ciliary roles in cortical development in mice lacking primary cilia as a consequence of mutations in either *Kif3a* or *Ift88* which encode components of the anterograde motor Kinesin II and the IFT-B complex, respectively. *NestinCre*;*Kif3a*^*fl/fl*^ conditional mutants in which *Kif3a* starts to be inactivated from embryonic day E10.5 in the dorsal telencephalon and in which cilia are lost by E13.5 showed an enlarged brain due to a shortening of the G1 phase of the radial glial cell cycle (Wilson et al., 2012). The increased cortical size may be primarily due to a reduced Gli3R formation. This idea is consistent with *Gli3*’s role in regulating G1 phase length by controlling the expression of the cell Cyclin dependent kinase 6 (*Cdk6*) (Hasenpusch-Theil et al., 2018). These findings suggest that primary cilia are essential as a negative regulator of RGC proliferation, a conclusion that was later confirmed by *Kif3a* knock-down experiments (Chen et al., 2019). In contrast, cilia abrogation was only complete by E14.5 in a different set of *NestinCre*;*Kif3a*^*fl/fl*^ mutants (Foerster et al., 2017). Interestingly, this delayed cilia inactivation did not lead to Gli3 processing defects but to an up-regulation of the mTORC1 pathway which in turn resulted in an augmented surface area of RGC apical endfeet and in an enlarged cortex. Taken together, these findings indicate that cilia have different, time-dependent roles in radial glial cells and may exert these functions by controlling different signaling pathways.

### Interference With Cilia Mediated Signaling

In addition to these studies on the complete loss of cilia, several other investigations determined the effects of manipulating cilia mediated signaling thereby in general observing more subtle effects on RGC behavior. These mutations affected neurogenic rather than proliferative divisions and led to subsequent changes in the relative proportions of cortical layers. *Gpr161* encodes an orphan G protein coupled receptor (GPCR) that acts as a negative regulator of Shh signaling by controlling cAMP levels and thereby Gli3 processing and repressor formation (Mukhopadhyay et al., 2013). Conditional inactivation of *Gpr161* during early cortical neurogenesis but not at mid-neurogenesis resulted in an increase in the number of basal progenitors and outer radial glial cells, a neural stem cell type rarely present in mice but predominant in the developing human cortex (Shitamukai et al., 2011; Wang et al., 2011). Consistent with this change, the formation of Tbr1^+^ deep layer neurons was decreased while the numbers of Satb2^+^ upper layer neuron was increased (Shimada et al., 2019). These findings indicate a strong requirement for cilia mediated repression of Shh signaling to enable normal cortical morphogenesis. The opposite effect, namely a down-regulation of basal progenitor and neuron formation, was observed in mice mutant for *Rpgrip1l* (Postel et al., 2019), which is crucial for establishing the transition zone (Mahuzier et al., 2012; Reiter et al., 2012; Shi et al., 2017; Wiegering et al., 2018) and for controlling proteasome activity and Gli3 processing at the ciliary base (Gerhardt et al., 2015). Mice mutant for the Joubert Syndrome gene *Inpp5e* (Bielas et al., 2009; Jacoby et al., 2009) displayed another neurogenic phenotype and preferentially generated cortical projection neurons at the expense of basal progenitors in early corticogenesis (Hasenpusch-Theil et al., 2020). This transient defect reflected an increased ratio of direct vs. indirect neurogenesis and resulted in the increased formation of Ctip2 + layer V neurons. The structure of the RGC cilium was severely affected thereby impairing Gli3 proteolytic processing and Gli3 repressor formation. Re-introducing the Gli3 repressor restored basal progenitor levels, though two copies of the *Gli3*^Δ699^ allele were required for a full rescue suggesting that reduced levels of Gli3R rather than a reduction in the Gli3R/Gli3FL ratio are responsible for the prevalence of direct neurogenesis (Hasenpusch-Theil et al., 2020). Taken together, the analyses of these three mouse mutants revealed diverse roles of cilia in controlling neurogenic divisions in the developing cortex thereby influencing the subtype composition of cortical projection neurons in varied and subtle ways. The basis for this remarkable variety in phenotypes, however, remains unknown. Elucidating this diversity requires a detailed, comparative analysis of pathway activities and gene expression changes in these mutants.

### Effects of Cilium Assembly and Disassembly on Cortical Neurogenesis

During the radial glial cell cycle, the primary cilium undergoes a cycle of assembly and disassembly and the timing of these events represents another critical factor for stem cell divisions. Constructing the primary cilium involves the formation of the distal appendages at the mother centriole and the docking of vesicles with cargo to build the cilium. On the other hand, cilia disassembly requires a multi-protein complex consisting of Aurora A, HDAC6, Nde1, and OFD1 (Pugacheva et al., 2007; Kim et al., 2011; Tang et al., 2013) that hereafter will be referred to as cilium disassembly complex. There is emerging evidence that interfering with these processes affects cortical stem cell behavior.

*Cep83* encodes a centrosomal protein that is crucial for the assembly of distal appendages leading to cilia initiation (Joo et al., 2013; Tanos et al., 2013). Its conditional inactivation using an *Emx1Cre* driver line, that mediates *Cre* expression in the dorsal telencephalon from E9.5, impaired the anchoring of the centrosome to the apical membrane of RGCs and the formation of the primary cilium (Shao et al., 2020). Due to an increased stem cell pool, these defects resulted in an enlarged cortex which formed a prominent fold in the cingulate cortex. Furthermore, centrosome detachment disrupted microtubule organization and led to a stretching and stiffening of the apical membrane and a concomitant increase in the expression of the Hippo co-transcriptional regulator Yap. Conditional removal of *Yap* in a *Cep83* mutant background rescues cortical size suggesting that *Cep83* controls cortical growth via Hippo signaling. However, Yap has a prominent role in suppressing ciliogenesis (Rausch and Hansen, 2020). Moreover, several mouse mutants with overexpression of Shh signaling (Yabut et al., 2015; Wang et al., 2016; Shimada et al., 2019) and *Gli3* conditional mutants (Amaniti et al., 2013) all phenocopy the enlarged cortex and the characteristic fold of the cingulate cortex in *Cep83* mutants. Taken together, these findings raise the possibility that the loss of primary cilia plays a role in the *Cep83* mutant cortical phenotype downstream of Yap activation. A careful time course analysis of Shh signaling and Gli3 processing could shed further light onto the involvement of cilia in the development of the *Cep83* phenotype.

In addition to cilia formation, the control of cilia disassembly is also critical in determining cortical growth, for example mutations in *Cenpj* or *centrosomal-P4.1-associated protein* (*CPAP*) cause dwarfism, microcephaly and intellectual disability (Al-Dosari et al., 2010) through a depletion of the cortical stem cell pool (Gabriel et al., 2016; Lin et al., 2020). Cenpj is thought to control RGC proliferation in two ways. Cenpj is required for centriole duplication and Cenpj mutations lead to misorientation of the mitotic spindle and widespread apoptotic cell death of RGCs (McIntyre et al., 2012; Lin et al., 2020). In addition, Cenpj acts as a scaffold for the cilium disassembly complex (Gabriel et al., 2016). Consequently, conditional removal of Cenpj resulted in microcephaly, longer primary cilia and abnormal cilium appendages in RGCs (Ding et al., 2019). While these findings left the relative contribution of abnormal centrioles and cilia to the cortical phenotype open, Gabriel et al. recently reported a novel splice site mutation in *CPAP* which removed the C-terminal coding exons. This mutation leaves CPAP centrosomal functions intact but interferes with its ability to interact with the cilium disassembly complex (Gabriel et al., 2016). In cortical organoids derived from patient specific iPSCs, RGCs undergo premature neurogenesis and have longer cilia, defects that were rescued by overexpression of the truncated part of the protein suggesting that CPAP predominately acts by controlling cilia disassembly in neuronal differentiation (Gabriel et al., 2016). This idea is consistent with the finding that *Nde1*, a component of the ciliary disassembly complex, also controls cortical growth. *Nde1* mutations can cause microcephaly and ciliary defects linking cilium disassembly with cell cycle progression (Kim et al., 2011). In addition, Nde1 has a function in orientating the mitotic spindle (Johnstone et al., 2019). Taken together, these examples provide emerging evidence that controlling the formation and dismantling of primary cilia might be crucial for regulating cortical stem cell behavior. However, they also illustrate that distinct roles of centrosomal or microtubule associated proteins in centrosome-associated process and in cilia assembly/disassembly and function can contribute to cortical growth control. In the future, dissecting these different contributions requires detailed protein interaction and genetic analyses.

## Primary Cilia and Neuron Migration

**FIGURE 3:**
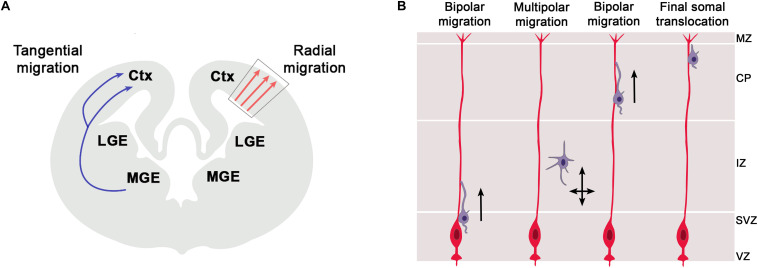
Radial and tangential migration of telencephalic neurons. **(A)** The cerebral cortex consists of two major classes of neurons, glutamatergic projection neurons and GABAergic interneurons which are generated in the cortex and ventral telencephalon, respectively. These neurons acquire their final position in the mature cerebral cortex by two different migratory routes. Cortical projection neurons migrate radially from the cortical ventricular zone into the cortical plate whereas cortical interneurons use tangential migration to enter the developing cortex from the ventral telencephalon. Within the cortex, these latter neurons initially continue on their tangential migration path along the marginal zone (MZ) or the intermediate zone (IZ) till they eventually change to radial migration to invade the cortical plate and to settle in their final position. **(B)** Radial migration of cortical projection neurons from the ventricular zone to their final position in the cortical plate. Newly born neurons migrate along the radial glial scaffold from the ventricular zone (VZ) into the subventricular zone (SVZ) where they detach from the glial process, assume a multipolar morphology and continue their migration in a RGC independent manner. Arriving at the intermediate zone (IZ), they transit to a bipolar morphology, re-attach to the glial scaffold, invade the cortical plate (CP) by glia-guided locomotion and bypass earlier formed neurons. In this way, cortical layers are formed in an inside-out manner with later born neurons acquiring positions in the upper cortical layers. Near the marginal zone (MZ), migrating neurons attach their leading process to the MZ and switch to glia-independent somal translocation. This step involves separate movements of nucleus and centrosome whereby the centrosome first moves into a swelling of the leading process followed by nucleokinesis.

In addition to their function in RGCs, there is an emerging role of primary cilia in the migration of newly born neurons to their final position in the cortical plate. The cerebral cortex consists of two major classes of neurons, glutamatergic projection neurons and GABAergic interneurons which are generated in the cortex and ventral telencephalon, respectively (Figure 3A). These neurons acquire their final position in the mature cerebral cortex by two different migratory routes. Cortical projection neurons migrate radially from the cortical ventricular zone into the cortical plate whereas cortical interneurons use tangential migration to enter the developing cortex from the ventral telencephalon. Primary cilia are pivotal to interneuron migration since disrupting ciliary functions in cortical interneurons interfered with the migratory process and led to abnormal positioning of interneurons in the adult cortex (Baudoin et al., 2012; Higginbotham et al., 2012). In contrast, much less is known about the involvement of cilia in radial migration of cortical projection neurons but recent studies start to elucidate some emerging roles and the underlying signaling mechanisms.

Radial migration of cortical projection neurons from the ventricular zone to their final position in the cortical plate is a complex, multistep process (Figure 3B). This initially involves migration along the radial glial scaffold and RGC independent migration in the subventricular zone (SVZ) (Tabata and Nakajima, 2003), followed by glia-guided locomotion (Noctor et al., 2004) and finally glia-independent somal translocation (Nadarajah et al., 2001; Franco et al., 2011). Whereas the cellular basis of these migratory processes are well understood, we only start to gain insights into the involvement of primary cilia. A subset of Meckel Gruber Syndrome and Joubert Syndrome patients develop neuronal migration defects including neuronal heterotopias where neurons accumulate in ectopic positions outside the cortical layers (Poretti et al., 2011; Adams et al., 2012). Such heterotopias were also present in mice mutant for *Gpr161* (Shimada et al., 2019) and in the Joubert genes *Rfx3* and *Inpp5e* (Magnani et al., 2015; Hasenpusch-Theil et al., 2020). The formation of periventricular heterotopias can also be caused by mutations in the *ADP-ribosylation factor guanine nucleotide-exchange factor-2* (*ARFGEF2*) and *Filamin A* genes (Fox et al., 1998; Sheen et al., 2004), with the latter controlling ciliogenesis in an *Arfgef2* dependent manner (Adams et al., 2012; Zhang et al., 2013). Moreover, mice carrying mutations in the *Eml1* gene encoding a microtubule-associated protein show subcortical heterotopias due to a mispositioning of radial glial cells and impaired primary cilia formation (Uzquiano et al., 2019). Finally, Guo et al. (2015) took a systematic approach and identified a panel of 17 ciliopathy genes knock-down of which resulted in a number of migration defects including a delayed multipolar to bipolar transition, an increase in leading process branching and/or a shortening of the leading process. Similarly, knock-down of *Ift172* encoding a component of the IFT-B complex, also resulted in a delayed migration of cortical projection neurons (Pruski et al., 2019). Taken together, these findings clearly suggest that primary cilia are important to radial migration of cortical projection neurons but their exact role(s) remained elusive from these initial studies.

Recently, Park et al. (2018) went a step further and characterized a role for primary cilia in the transition from multipolar to bipolar morphology occurring at the arrival of radially migrating neurons in the intermediate zone. Focal malformations of cortical development (FMCDs) are a group of disorders that are highly associated with medically intractable epilepsy, intellectual disability, developmental delay, and autism-spectrum disorders and are characterized by neuronal migration defects leading to cortical dyslamination (Foldvary-Schaefer et al., 2004; Wegiel et al., 2010; Lim and Crino, 2013). A major cause of these malformations are somatic mutations in MTOR, an evolutionarily highly conserved serine/threonine kinase and a central regulator of the mTOR pathway, leading to an increase in mTOR signaling and a concomitant decrease in autophagy. In their recent publication, Park et al. (2018) used *in utero* electroporation of MTOR constructs carrying patient specific mutations to mimic the disease. This interference resulted in a delay of neuronal migration with a large proportion of neurons remaining in the intermediate zone where they failed to transit from multipolar to bipolar morphology. Interestingly, ciliogenesis was disrupted in electroporated neurons as well as in neurons in brain samples from FMCD patients raising questions how mTOR signaling controls cilia formation and in turn how cilia regulate the migration of projection neurons. Based on elegant and systematic analyses, the authors proposed a model in which up-regulation of mTOR signaling resulted in a decrease in autophagy and thereby in the accumulation of OFD1 protein, a negative regulator of cilia formation (Singla et al., 2010; Tang et al., 2013), at the centriolar satellites. These ciliary defects subsequently disturb a fine-tuned balance between canonical and non-canonical Wnt signaling which underlies the multipolar to bipolar transition of migrating neurons at the IZ (Boitard et al., 2015). These detailed analyses provide a convincing, first link between ciliary defects caused by increased mTOR signaling and defective cortical migration.

Park et al. (2018) also observed that many mTOR overexpressing neurons eventually leave the intermediate zone but do not reach their final position in the upper cortical layers. Hence, it is possible that defective cilia affect later steps in cortical neuron migration such as glial-guided locomotion and somal translocation. During this latter process, the centrosomes are coupled with the nucleus and this coupling allows nucleokinesis (Tanaka et al., 2004; Bellion et al., 2005; Tsai et al., 2007; Belvindrah et al., 2017). Given the close link between cilia and the centrosome, it is highly likely that primary cilia regulate this migratory mode. This idea has recently been studied in two papers investigating the migration of neuroblasts from the subventricular zone along the rostral migratory stream (RMS) into the olfactory bulbs. Matsumuto et al. (2019) first confirmed the presence of primary cilia on migrating neuroblasts in several vertebrate species. A combination of live imaging and detailed ultrastructural analyses further revealed a surprisingly dynamic localization and orientation of the primary cilium depending on the mitotic state and the migration step. Neuroblasts can still divide during their migration and suppress ciliogenesis during mitosis but cilia formation is promoted after cell cycle exit. Initially, the cilium is submerged in the cytosol with a posterior orientation when neuroblasts extend their leading process. However, it is extended from the cell surface and moves forward within the swelling but submerges again during nucleokinesis. This highly organized dynamics of cilium maturation and positioning is pivotal for neuroblast migration as interfering with *Kif3a* or *Ift88* function impaired both ciliogenesis and neuroblast movement (Matsumoto et al., 2019). An investigation by Stoufflet et al. (2020) recently confirmed this dependence on primary cilia function and provided insights into the underlying mechanism (Stoufflet et al., 2020). These authors analyzed the distribution of cAMP in migratory neuroblasts and identified a cAMP rich region (hotspot) that was located at the centrosome and emanated from the primary cilium. Conditional inactivation of *Kif3a* or *Rpgrip1l*, or knock-down of ciliary *Adenylate cyclase 3* (*AC3*) abolished hotspot formation and resulted in defective centrosome dynamics and altered nucleokinesis. Interfering with the centrosomal localization of PKA phenocopied these migratory defects strongly suggesting a direct link between the primary cilium and the centrosome through cAMP signaling. Interestingly, the authors found a similar cAMP hotspot in migrating cortical projection neurons. While this raises the interesting possibility that cAMP signaling is similarly involved in somal translocation of cortical projection neurons, it remains to be seen whether other signaling pathways and in particular Reelin signaling which is critical for correct cortical layering (Tissir and Goffinet, 2003) also control migratory behavior of cortical projection neurons via the cilium. Such future studies may provide important insights into the pathomechanisms of ciliopathies with a neurological component where migration defects may contribute to the severity of the illness.

## Primary Cilia and Axon Pathfinding in the Cerebral Cortex

One of the best characterized roles of primary cilia in corticogenesis relates to their function in the development of the corpus callosum, the largest fiber tract in the brain connecting the two cerebral hemispheres. Malformations of the corpus callosum are a hallmark of ciliopathies (Tobin and Beales, 2009) and have been identified in Joubert (Poretti et al., 2011), Meckel Gruber (Salonen, 1984), Acrocallosal (Odent et al., 1998; Holub et al., 2005; Takanashi et al., 2009; Putoux et al., 2011), and Orofacialdigital Syndrome patients. They can manifest as partial agenesis, hypoplasia across the entire structure or complete agenesis (ACC).

**FIGURE 4:**
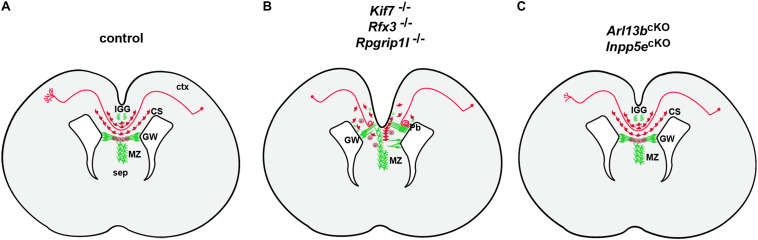
Control of control corpus callosum development by primary cilia. **(A)** Formation of the corpus callosum in control embryos. Initially, callosal neurons extend an axon either toward the corpus callosum to connect with neurons on the contralateral side. Reaching the boundary between cortex (ctx) and septum (sep) callosal axons cross from one cerebral hemisphere to the opposite (midline crossing). This crossing is controlled by the midline glial cell populations of the glial wedge (GW), indusium griseum (IGG) and midline zipper glia (MZ) in combination with neurons of the callosal sling (CS). These cells occupy strategic positions along the path of callosal axons and act as guideposts to channel these axons across the midline by expressing specific axon guidance molecules (Lindwall et al., 2007; Paul et al., 2007). Once in their target region, these axons heavily branch to innervate target neurons. **(B)** In *Kif7*^–/–^, *Rfx3*^–/–^, and *Rpgrip1l*^–/–^ mutants, midline glia and callosal sling neurons are severely disorganized with glial fibers extending between the ventricular and pial surfaces block the path of callosal axons which form Probst bundles (Pb). **(C)** In *Arl13b*^*cKO*^ and *Inpp5e*^*cKO*^ mutants, callosal axons cross the midline but display reduced branching and innervation of target layers.

The analyses of several ciliary mouse mutants revealed a requirement for cilia in multiple steps of callosal development (Figure 4A). In the previously mentioned *Inpp5e* mouse mutant, callosal axons cross to the contralateral hemisphere but the corpus callosum is thinner. This defect has been attributed to a decrease in Gli3 repressor formation resulting in an expansion of the piriform cortex at the expense of the neocortex and a concomitant reduction in callosal projection neurons (Hasenpusch-Theil et al., 2020). Hence, the authors suggested that the *Inpp5e* mutation affects the initial specification of callosal projection neurons through a patterning defect. Indeed, crossing *Inpp5e* mutants with *Gli3*^Δ699^ animals that can only form the Gli3 repressor restored Gli3 repressor levels and the size of both the neocortex and the corpus callosum (Hasenpusch-Theil et al., 2020). While *Inpp5e* mutants display a relatively mild callosal phenotype, other cilia mouse mutant show more severe malformations with callosal axons failing to cross the midline. In an *in utero* electroporation assay, knock-down of several ciliary genes including *Bbs5*, *Bbs7*, *Bbs11*, *Bbs12*, and *Kif7* resulted in reduced midline crossing and *Bbs5* and *Bbs7* deficient axons aberrantly projected into the septum (Guo et al., 2015) but the underlying mechanisms were not further investigated. Detailed mechanistic insights into the role of primary cilia in midline crossing came from the analyses of *Rfx3*, *Rpgrip1l*, and *Kif7* mutant mice (Benadiba et al., 2012; Laclef et al., 2015; Putoux et al., 2019). *Rfx3* encodes a transcription factor controlling the expression of many genes involved in ciliary assembly and function (Thomas et al., 2010); *RPGRIP1L* is mutated in Joubert and Meckel Gruber Syndromes (Arts et al., 2007; Delous et al., 2007) and codes for a transition zone protein. *KIF7* mutations were identified in Acrocallosal and Joubert Syndromes (Dafinger et al., 2011; Putoux et al., 2011) and *Kif7* plays critical roles in regulating Sonic hedgehog signaling by organizing the ciliary tip compartment (He et al., 2014). All three mouse mutants are characterized by complete agenesis of the corpus callosum with callosal axons forming large Probst bundles, aberrant bundles of axons that run longitudinally along the rostrocaudal axis rather than crossing the midline (Benadiba et al., 2012; Laclef et al., 2015; Putoux et al., 2019). Moreover, the distribution of neuronal and glial guidepost cells which steer callosal axons toward the contralateral hemisphere was severely disturbed in these mutants (Figure 4B). While there were slight differences in the extent and type to which guideposts were affected, elegant transplantation experiments in *Rfx3* and *Rpgrip1l* mutants revealed that the primary defect resides in the mislocalization of these guideposts rather than in a misspecification of callosal projection neurons (Benadiba et al., 2012; Laclef et al., 2015). In these experiments, mutant axons were capable of midline crossing in a control environment while conversely control axons failed to cross the midline in a mutant setting raising the question how cilia control the formation of an environment conducive to callosal axon migration toward the contralateral side. A first clue to the origin of the midline defects came from a comparison with *Gli3* mouse mutants that not only displayed ACC but also a very similar disorganization of callosal guideposts (Amaniti et al., 2013; Magnani et al., 2014). This disorganization in *Gli3* mutants resulted from defective patterning of the cortical/septal boundary, where callosal axons cross to the contralateral side. Similar defects at this boundary were subsequently identified in the three ciliary mutants (Benadiba et al., 2012; Laclef et al., 2015; Putoux et al., 2019). Moreover, the observation that *Rfx3*, *Rpgrip1l*, and *Kif7* mutants showed a reduced formation of the Gli3 repressor further corroborated the involvement of Gli3 (Benadiba et al., 2012; Laclef et al., 2015; Putoux et al., 2019). Most strikingly, however, crossing *Rfx3*, *Rpgrip1l*, and *Kif7* mutants with *Gli3*^Δ699^ repressor mice completely rescued the severe callosal malformation (Laclef et al., 2015; Putoux et al., 2019). Taken together, these findings indicate that primary cilia play a critical role in midline crossing of callosal axons by controlling the placement of guidepost cells at the cortical/septal boundary through Gli3 repressor formation at patterning stages.

In addition to these findings demonstrating a cell non-autonomous role for cilia, a recent publication revealed that primary cilia can also act in neurons cell autonomously to control callosal formation (Guo et al., 2019). These authors used conditional inactivation of the Joubert genes *Arl13b* and *Inpp5e* in callosal projection neurons which is predicted not to affect midline organization. They found that mutant axons were poorly fasciculated, formed a wider CC tract and their growth cones displayed longer filopodia. Reaching the contralateral hemisphere, *Arl13b*-and *Inpp5e*-deficient axons displayed reduced axonal branching and innervation of target layers (Figure 4C). These findings were even more astonishing if one considers that the cilium extends from the neuronal cell body which is far apart from the axonal growth cone raising the question how cilia can affect axon guidance and synapse formation across such large distances. The authors addressed this question with a sophisticated approach using a combination of chemogenetics and optogenetics to express and activate signaling components specifically in the primary cilium of cultured neurons. They first showed that *Arl13b* deficiency disrupts the ciliary localization of its downstream effector Inpp5e leading to increased PIP3 levels and AKT activity in the cilium. Interestingly, increased AKT signaling spread into the growth cone and rapidly caused a retraction of lamellipodia and a concomitant extension of filopdia. Similar effects were observed after cilia specific activation of AKT or PI3K whereas activation of the Inpp5e phosphatase had opposite consequences on growth cone dynamics. Finally, inhibition of actin polymerization abolished the growth cone changes induced by ciliary PI3K/AKT activation. These findings suggests the existence of a self-perpetuating feedback network between AKT signaling and actin polymerization that rapidly spreads local signaling over long distances along the plasma membrane to reach distant cellular targets (Sawano et al., 2002; Kholodenko, 2006; Fivaz et al., 2008; Welf et al., 2012; Katsura et al., 2015; Guo et al., 2019).

Overall, these analyses of several mouse mutants provide a framework for understanding how defects in cilia controlled processes underlie agenesis of the murine corpus callosum but also emphasize the need to investigate mechanisms of corpus callosum formation in humans where the corpus callosum extends along the nearly complete rostrocaudal axis of the cortex. It also remains open whether other forebrain axon tract like the thalamocortical tract which relays sensory information from the thalamus to the cortex and which is defective in ciliary mouse mutants (Magnani et al., 2015) are also affected in ciliopathy patients.

## Primary Cilia and Neuronal Circuit Formation

A number of ciliopathies are associated with severe neurological features including ASD, Intellectual Disability (ID) and epilepsy (Valente et al., 2014). A major cause of these disorders are thought to be abnormalities in synapse formation and function thereby suggesting that primary cilia may play a role in neuronal circuit formation. While such ciliary functions have only recently been started to be investigated, some important roles are emerging from these studies. Overexpression of a ciliary targeted 5HT6 receptor in cultured cortical neurons resulted in increased cilia length, reduced AC3 levels and disrupted dendrite growth and arborization (Guadiana et al., 2013). These effects were reversed by interfering with cilia formation and by overexpression of AC3 suggesting that dendritogenesis in cultured neurons requires cilia mediated AC3 signaling. Guo et al. (2019) extended these findings and revealed an *in vivo* role for ciliary signaling in axon and dendrite development. As mentioned previously, conditional inactivation of *Arl13b* or *Inpp5e* in callosal projection neurons resulted in a wider corpus callosum with poorly fasciculated callosal axons (Guo et al., 2019). Arriving at their target areas in the contralateral hemisphere, these axons also had significantly reduced terminal arbors which are crucial for establishing connections with target neurons (Gibson and Ma, 2011; Kalil and Dent, 2014). In addition, the axonal shafts of mutant neurons showed less branching, which allows projecting axons to connect with additional targets en route (Guo et al., 2019). These findings strongly suggest that *Arl13b* and *Inpp5e* deficiency leads to reduced innervations of their target areas. It is certainly tempting to speculate that these malformations result from defective spreading of signals from the cilium located at the neuronal soma toward the tip of the axon as described for filopodia and lamellipodia formation in cultured neurons but this still needs to be shown. Taken together these studies make it very likely that ciliopathy mutations that disrupting ciliogenesis and/or ciliary signaling may affect dendritogenesis and therefore the ability of neurons to form normal network connections and brain circuitry (Guadiana et al., 2013).

In models, single neurons are often depicted with their primary cilium protruding from the surface of the neuronal soma at a distance from dendrites and synapses. However, projection neurons and interneurons are very densely packed in the developing and adult cortex and as such cilia will be in close contact with other neurons, their dendrites and axons. This raises the interesting possibility that due to their position cilia represent excellent candidates as modifiers of neuronal circuitry. This certainly is an area much to learn about in the developing cerebral cortex but we are starting to gain insights into ciliary roles in other forebrain neurons in shaping neuronal connectivity. In addition to cortical interneurons, the medial ganglionic eminence generates striatal interneurons that regulate the activity of medium spiny neurons in the striatum and thereby striatal output (Gittis et al., 2010). It was recently shown that *Arl13b* conditional inactivation in interneurons caused alterations in interneuron morphology and electrophysiological properties with reductions in synaptic buttons density and synaptic size (Guo et al., 2017). Moreover, activation of G protein coupled receptor (GPCR) signaling or induction of the Somatostatin receptor 3 (SstR3) rescued these morphological and synaptic defects. Thus, these findings indicate a mechanism by which cilia can influence circuit formation and function in a synapse independent manner. Given that a variety of GPCRs and other neuropeptide and neurotrophin receptors with important neurological functions (Berbari et al., 2008; Domire et al., 2011; Green et al., 2012; Guadiana et al., 2016; Bowie and Goetz, 2020) accumulate in primary cilia, this organelle has the potential to act as a modifier of neuronal circuitry. Characterizing such roles in the cerebral cortex where cilia could not only play a role in cortical interneuron migration but also in establishing and shaping interneuron connections with glutamatergic projection neurons will be of great importance for understanding the pathomechanims of many neurodevelopmental disorders in which an imbalance between cortical excitation and inhibition has been identified as a common cause (Bourgeron, 2009; Marin, 2012).

## Outlook and Concluding Remarks

In this review, we have summarized our current knowledge on the role of primary cilia during corticogenesis. This tiny organelle plays multiple and remarkably varied roles at various steps of cortical development. A major focus in the future will be to elucidate how a combination of defects in ciliary controlled signaling pathways contribute to this variability in phenotypes. Throughout this review, we have emphasized the importance of ciliary mediated processing of Gli3 for cortical patterning and stem cell development reflecting a strong requirement to suppress Hedgehog signaling in these early steps of corticogenesis. In contrast, Hedgehog signaling becomes increasingly more active at later stages to control the formation of olfactory bulb interneurons and of glial cells (Zhang et al., 2020) and establishing and maintaining the neurogenic niche in the SVZ (Petrova et al., 2013; Wang et al., 2014). It will be interesting to see whether cilia also control these processes in late corticogenesis. It is also important to note that while there are many commonalities between human and mouse cortex, human cortex formation differs in several aspects, most notably with respect to neural stem cell types and their proliferative behavior. As one example, outer radial glial cells are present in human cortex in much larger numbers than in mice and are thought to be responsible for the expansion of the human cortex during evolution. Their morphology, migration and proliferation is controlled by several signaling pathways linked to cilia including Shh and mTORC1 signaling (Andrews et al., 2020; Matsumoto et al., 2020) raising the possibility that cilia regulate the characteristics of these cells. Given such differences between human and mouse corticogenesis, it will be important to model ciliary functions in human corticogenesis in 2D cultures of neural stem cells or in human 3D cortical organoids. These analyses will also bring us a step closer toward elucidating the pathomechanisms underlying ciliopathies with neurological components and neurodevelopmental disorders that are not regarded as classical ciliopathies but for which a prominent role of primary cilia is emerging.

## Author Contributions

KH-T and TT conceived the idea and wrote the manuscript. Both authors contributed to the article and approved the submitted version.

## Conflict of Interest

The authors declare that the research was conducted in the absence of any commercial or financial relationships that could be construed as a potential conflict of interest.
